# COVID-19 among health workers in Brazil: The silent wave

**DOI:** 10.7189/jogh.10.010379

**Published:** 2020-06

**Authors:** Emanuelle Pessa Valente, Lia Cruz Vaz da Costa Damásio, Leonardo Sérvio Luz, Marília Francisca da Silva Pereira, Marzia Lazzerini

**Affiliations:** 1 Institute for Maternal and Child Health – IRCCS “Burlo Garofolo”, Trieste, Italy; 2 Universidade Federal do Piauí – UFPI, Teresina, Piauí, Brazil

According to the most recent WHO estimates, Brazil has the highest number of diagnosed COVID-19 cases in the Americas Region after the United States [1]. Community transmission has been documented throughout all federal units (26 states and Federal district).

Since the beginning of pandemic, many organizations have raised concerns with the lack of personal protective equipment (PPE), low observance of social distancing measures, and scarce availability of diagnostic tests in Brazil [2,3]. MoH recommended use of diagnostic swabs be reserved for severe cases with Acute Respiratory Distress Syndrome (ARDS) [4]. No specific federal recommendations on case finding among health workers (HW) currently exist.

Data from other countries have clearly indicated that HW are disproportionally affected by COVID-19 and can be carriers of the disease. In Italy, 20 618 COVID-19 cases have been reported so far among HW (10.4% of total cases) [5]. The Italian National Federation of Medical Doctors and Odontologists has reported 151 deaths among doctors [6]. These data do not include other HW categories such as nurses or midwives. In US, the Centers for Disease Control and Prevention (CDC) reported 9282 COVID-19 cases confirmed among HW [7] among these 723 (8%-10%) were hospitalized and 184 (2%-5%) required intensive care unit (ICU) admission.

In defense of HW safety, the Brazilian Federal Council of Medicine (FCM) has taken several measures. HW safety guidelines have been widely circulated, with hospital inspections carried out to verify their implementation. An online platform has been established for professionals to report shortcomings of resources, such as lack of PPE in workplaces, either public or private. Finally, the FCM is advocating for expanding criteria for COVID-19 diagnostic tests to all symptomatic HW [8].

Yet frontline workers are dangerously ill-equipped due to decades of underinvestment in the public health sector and limited access to appropriate PPE and training [9,10]. The Brazilian Federal Council of Nursing highlighted around 4800 reports of lack of PPE made by associate members since the beginning of pandemic. In the same time period, there have been more than 4600 sick leaves for “influenza-like symptoms” and 32 deaths among nurses, numbers significantly higher than usual trends [10]. Brazilian media have claimed that the number of COVID-19 cases and related deaths among HW, in particular in selected states such as São Paulo and Maranhão, is rapidly increasing [11].

**Figure:**
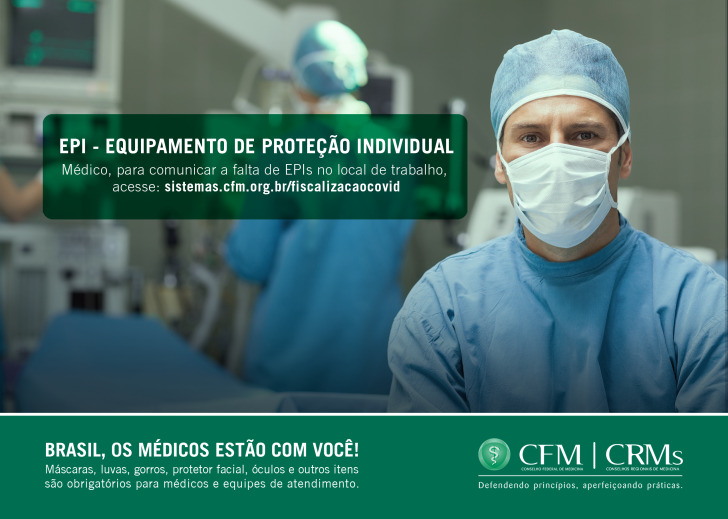
Photo: Brazilian Federal Council of Medicine online platform for reporting shortcomings of resources (eg, personal protective equipment) in workplaces. Used with permission kindly provided by Brazilian Federal Council of Medicine.

Open Knowledge Brazil (OKBR), a civil society organization that operates in support of open-access data of public interest, ranked Brazilian states with a “Transparency index”, evaluating 13 criteria related to content, format and level of detail of information disclosed via official portals during COVID-19 pandemic [12]. Despite improvements in the last weeks, on 22 April only four (15.3%) Brazilian states published data on the availability of COVID-19 diagnostic tests, while 11 (42.3%) provided data on incidence of new ARDS cases [12]. The “Transparency index” had a major impact on public opinion in Brazil, and civil public legal action was taken against San Paulo state using these data. However, the Transparency index does not include availability of data on COVID-19 among HW to evaluate states.

We report here the results of a rapid review performed by systematically screening each of the 27 federal health department websites and COVID-19 dedicated portals in order to identify specific policies for HW health screening and testing, and related HW morbidity and mortality data. Data collection procedures were integrated by research on social networks. Data are updated on 27 April 2020.

Results indicated that Pernambuco, a state in Northeast, was the first to develop a policy to perform diagnostic swabs among all symptomatic HW on 4 April 2020, giving priority to HW in ICUs and emergency departments. Policies in other states were less clear, with limited availability on official websites. Major investments were made in rapid tests for qualitative antibody detection whose accuracy is still unclear.

**Figure 1:**
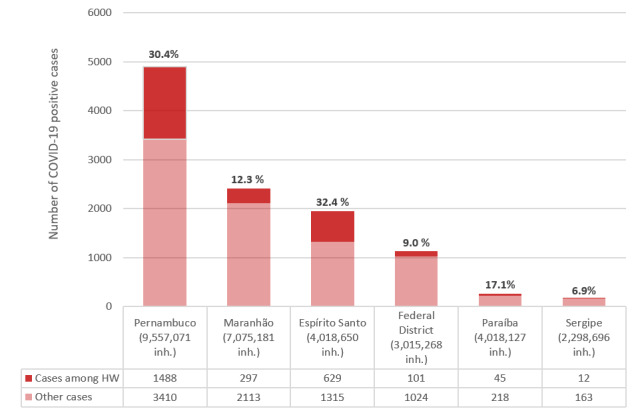
COVID-19 positive cases among health workers by Brazilian federal state. HW – health worker. Note: only six states had data available on health worker infection; Pernambuco state has a policy for HW testing. Data sources: State epidemiological bulletins, accessed 27 April 2020 [13-19].

Information regarding COVID-19 confirmed cases among HWs was available in the official bulletins of only six (22.2%) Brazilian Federal states (**Figure 1**). As expected based on current policies, a significantly higher number of cases was detected in Pernambuco compared to other states, with a high prevalence in HW (30.8% of total cases). As many states are currently implementing massive rapid test programs, increased numbers of COVID-19 cases among HW are expected in coming weeks.

These data demonstrate a lack of a homogeneous, transparent, and comprehensive surveillance system for COVID-19 cases among Brazilian HW during the current pandemic. Coordinated policies are needed to increase HW protection, and availability of surveillance data, to protect both HW and the entire Brazilian population.
